# A rare case of secondary syphilis

**DOI:** 10.11604/pamj.2022.42.224.35926

**Published:** 2022-07-21

**Authors:** Surya Besant Natarajan, Krishna Prasanth Baalann

**Affiliations:** 1 Department of Community Medicine, Sree Balaji Medical College and Hospital, Bharath Institute of Higher Education and Research, Chennai, India

**Keywords:** Wartlike sores, *treponema pallidum*, syphilis, IgG antibody, agglutination

## Image in medicine

The organism *Treponema pallidum* causes syphilis, a chronic inflammatory disease. Secondary syphilis is distinguished by the appearance of a wart-like sore 2 to 6 weeks after the chancre forms and, in some cases, before the chancre heals. The presence of other systemic symptoms indicates that the infection has spread to other parts of the body. During the secondary stage, a person is highly contagious. A 38-year-old man presented at the outpatient department with complaints of fever, weight loss patchy hair loss, and sore throat for the past five weeks. Physical examination revealed diffuse lymphadenopathy, hepatosplenomegaly, and the presence of reddish wartlike sores over the glans penis. Blood investigations showed a positive venereal disease research laboratory test (VDRL). *Treponema pallidum* particle agglutination assay (TP-PA) was positive for *T. pallidum* specific immunoglobulin G (IgG) antibody. For treatment, a stat dose of 2g of azithromycin was given orally and was advised for follow-up. Sexual contact of syphilis patients should be evaluated.

**Figure 1:**
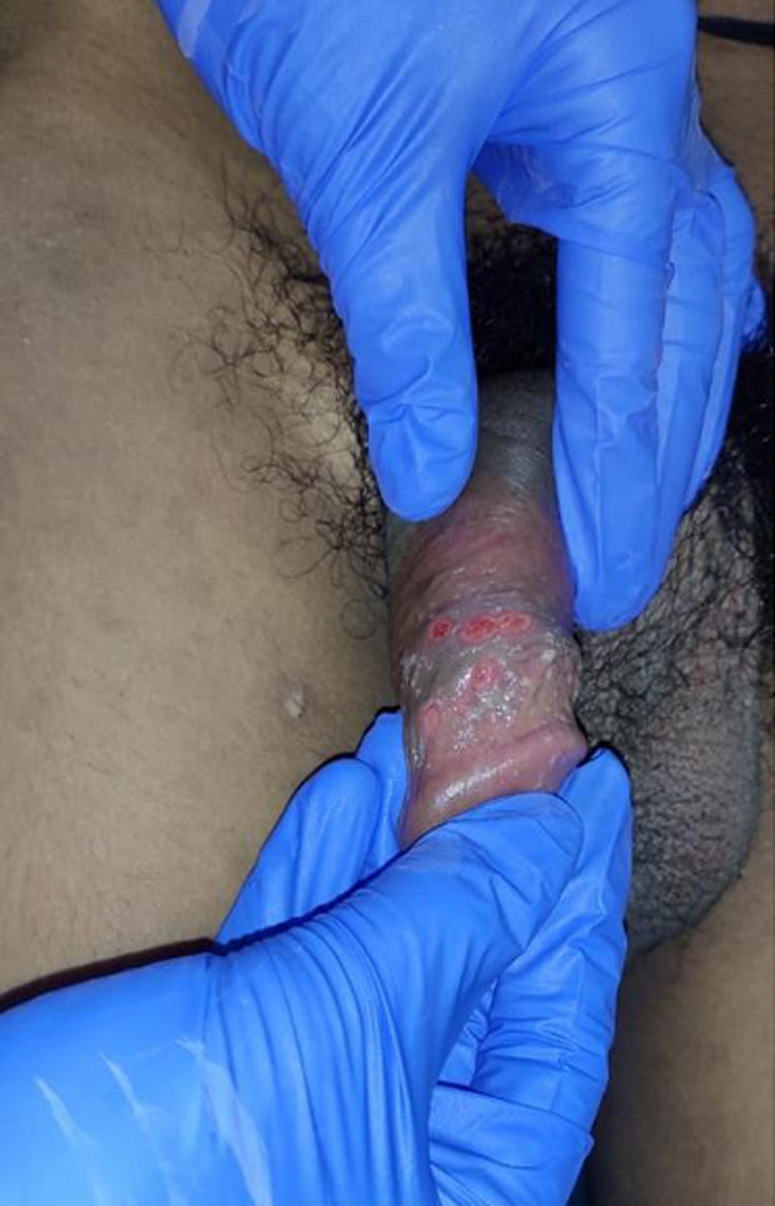
reddish wartlike sores over the glans penis

